# Lung Cancer Segmentation With Transfer Learning: Usefulness of a Pretrained Model Constructed From an Artificial Dataset Generated Using a Generative Adversarial Network

**DOI:** 10.3389/frai.2021.694815

**Published:** 2021-07-16

**Authors:** Mizuho Nishio, Koji Fujimoto, Hidetoshi Matsuo, Chisako Muramatsu, Ryo Sakamoto, Hiroshi Fujita

**Affiliations:** ^1^ Department of Diagnostic Imaging and Nuclear Medicine, Kyoto University Graduate School of Medicine, Kyoto, Japan; ^2^ Department of Radiology, Kobe University Hospital, Kobe, Japan; ^3^ Department of Real World Data Research and Development, Kyoto University Graduate School of Medicine, Hikone, Japan; ^4^ Faculty of Data Science, Shiga University, Gifu, Japan; ^5^ Preemptive Medicine and Lifestyle-Related Disease Research Center, Kyoto University Hospital, Kyoto, Japan; ^6^ Department of Electrical, Electronic and Computer Engineering, Faculty of Engineering, Gifu University, Gifu, Japan

**Keywords:** lung cancer, lung nodule, segmentation, computed tomography, deep learning, generative adversarial network 3

## Abstract

**Purpose:** The purpose of this study was to develop and evaluate lung cancer segmentation with a pretrained model and transfer learning. The pretrained model was constructed from an artificial dataset generated using a generative adversarial network (GAN).

**Materials and Methods:** Three public datasets containing images of lung nodules/lung cancers were used: LUNA16 dataset, Decathlon lung dataset, and NSCLC radiogenomics. The LUNA16 dataset was used to generate an artificial dataset for lung cancer segmentation with the help of the GAN and 3D graph cut. Pretrained models were then constructed from the artificial dataset. Subsequently, the main segmentation model was constructed from the pretrained models and the Decathlon lung dataset. Finally, the NSCLC radiogenomics dataset was used to evaluate the main segmentation model. The Dice similarity coefficient (DSC) was used as a metric to evaluate the segmentation performance.

**Results:** The mean DSC for the NSCLC radiogenomics dataset improved overall when using the pretrained models. At maximum, the mean DSC was 0.09 higher with the pretrained model than that without it.

**Conclusion:** The proposed method comprising an artificial dataset and a pretrained model can improve lung cancer segmentation as confirmed in terms of the DSC metric. Moreover, the construction of the artificial dataset for the segmentation using the GAN and 3D graph cut was found to be feasible.

## Introduction

Segmentation of lung cancer is an important research topic, and various studies have been conducted so far. Segmentation results are used to determine the effectiveness of anticancer drugs (Mozley et al., 2012; Hayes et al., 2016) and to perform texture analyses on medical images (Bashir et al., 2017; Yang et al., 2020). To use the segmentation results of lung cancer effectively, the segmentation accuracy is an important factor. Segmentation is typically done manually by radiologists; however, manual segmentation can sometimes yield inaccurate results because of interobserver variability. Semiautomatic segmentation has lower interobserver variability than manual segmentation (Pfaehler et al., 2020). To overcome this interobserver variability, an automatic segmentation of lung cancer is desirable.

Recent years have witnessed significant development in the application of deep learning to various domains, including in the area of segmentation. For example, deep learning has been applied to the automatic segmentation of organs, such as the lungs, liver, pancreas, uterus, and bones, and to the automatic segmentation of tumors in these organs, with good segmentation performance (Roth et al., 2015; Chlebus et al., 2018; Isensee et al., 2018; Chen et al., 2019; Gordienko et al., 2019; Kurata et al., 2019; Noguchi et al., 2020; Hodneland et al., 2021).

One of the problems in the application of deep learning is a dataset. Deep learning does not perform well when the dataset is small. In general, it is difficult to increase the size of datasets containing medical images compared with other domains. This is due to the high cost of acquiring medical images and the need to protect personal information. To this end, transfer learning with pretrained models (Shin et al., 2016; Tschandl et al., 2019), data augmentation (Zhang et al., 2017; Yun et al., 2019), and artificial generation of datasets using generative adversarial networks (GANs) (Muramatsu et al., 2020) have been developed.

The GAN was first proposed by Goodfellow et al. (2014). The recent improvements made to the GAN have made it possible to generate high-quality, high-resolution images. Various attempts have been made to apply the GAN to medical image processing. Several studies have shown that it is possible to generate CT images of lung nodules using the GAN (Jin et al., 2018; Han et al., 2019; Onishi et al., 2019; Yang et al., 2019; Yi et al., 2019; Armanious et al., 2020).

To overcome the small dataset problem for segmentation, we proposed to use deep learning models pretrained with an artificially generated dataset using the GAN. We hypothesized that transfer learning with the proposed pretrained models could improve the automatic segmentation accuracy when using the lung cancer dataset. In general, a segmentation model obtained through supervised learning requires an image and its label as the dataset. In our study, to generate a dataset for segmentation, we used the GAN for image generation and the 3D graph cut method for generating labels of the generated images. No manual task for labeling was required to generate the dataset for pretraining.

## Materials and Methods

**FIGURE 1:**
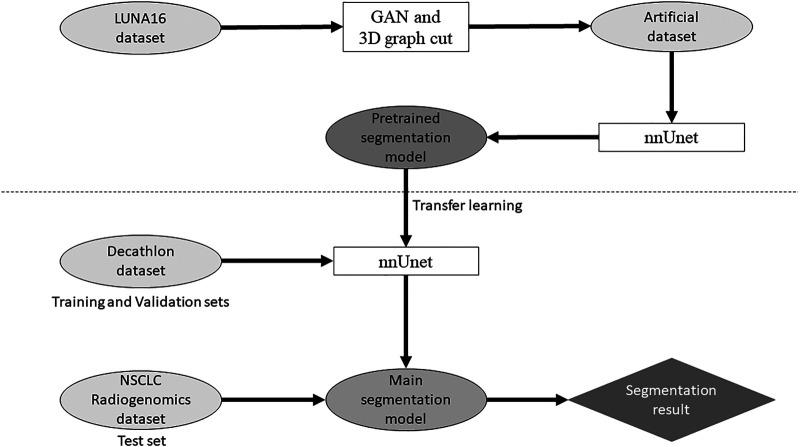
Outline of the proposed method for the segmentation model.

Our study used anonymized data extracted from public databases. Therefore, institutional review board approval was waived in accordance with the regulations of our country. Figure 1 shows the outline of the proposed method for the segmentation model.

### Dataset

Three public datasets containing computed tomography (CT) images were used: LUng Nodule Analysis 2016 (LUNA16) dataset, Decathlon lung dataset, and NSCLC radiogenomics. Table 1 shows a summary of the three datasets.

**TABLE 1:** Summary of three datasets.

Dataset	Number of all CT scans	Number of CT scans used in this study	Selection criteria	Usage of dataset
LUNA16	888	165	3D CT images with small lung nodules (the size of each nodule was <6 mm) were selected	From the LUNA16 dataset, the GAN generated an artificial dataset with generated lung nodules. The pretrained model for lung cancer segmentation was obtained from the artificial dataset with original nnUnet.
Decathlon (task06, lung)	63	■ 63 (Decathlon_full_) ■ 30 (Decathlon_mid_) ■ 10 (Decathlon_small_)	For Decathlon_full_, no selection criteria. The image files of the Decathlon dataset were sorted by file name, and the first 10 or 30 files were selected for Decathlon_small_ and Decathlon_mid_	Decathlon dataset was used for training and validation sets of lung cancer segmentation. Modified nnUnet was trained based on the validation set of the Decathlon dataset and the pretrained model.
NSCLC radiogenomics	211	144	Segmentation labels are available	NSCLC radiogenomics was used for test set of lung cancer segmentation (not used for the validation set).

Abbreviations: GAN, generative adversarial network.

The LUNA16 dataset includes 888 sets of 3D CT images (Grand-Challenges, 2016; Setio et al., 2017) constructed for lung nodule detection. Therefore, the original LUNA16 dataset is unsuitable for segmentation. A previous study used the LUNA16 dataset to generate images of lung nodules using the GAN (Nishio et al., 2020a). We used the same dataset and a GAN model to generate the dataset for segmentation. For image preprocessing, the voxel size of the 3D CT images in the LUNA16 dataset was changed (1 mm × 1 mm × 1 mm isotropic). To generate lung cancer–like nodules and their labels in the LUNA16 dataset, large true nodules are problematic because labels of true nodules are not available in the LUNA16. Therefore, sets of 3D CT images with small lung nodules (the size of each nodule was <6 mm) were selected. As a result, 165 sets of 3D CT images in the LUNA16 dataset were used to generate an artificial dataset for segmentation.

The Decathlon challenge (http://medicaldecathlon.com/) was held to provide a fully open source and comprehensive benchmark for general purpose algorithmic validation and testing, covering several segmentation tasks. Decathlon includes several segmentation datasets, from which the Decathlon lung dataset (Task06) was used as the training and validation sets for our study. The Decathlon lung dataset includes 63 sets of 3D CT images and their segmentation labels. To simulate the small dataset, 10 and 30 sets of 3D CT images were selected from the Decathlon lung dataset; the image files of Decathlon lung dataset (NIfTI files) were sorted by file name, and the first 10 or 30 files were selected. As a result, three types of training datasets were prepared from the Decathlon lung dataset: 63 sets from the original Decathlon lung dataset (Decathlon_full_), 30 sets (Decathlon_mid_), and 10 sets (Decathlon_small_). No image preprocessing was performed on the Decathlon lung dataset.

The NSCLC radiogenomics dataset (https://wiki.cancerimagingarchive.net/display/Public/NSCLC-Radiomics) contains images from 211 patients with non–small-cell lung cancer (Cancer Imaging Archive, 2021; Bakr et al., 2018; Gevaert et al., 2012; Clark et al., 2013). The dataset comprises CT, positron emission tomography/CT images, and segmentation maps of tumors in the CT scans. From the 211 patients, 3D CT images of 144 patients and their segmentation labels were selected for the current study. Segmentation labels are not available for the other 67 patients. The NSCLC radiogenomics dataset was used as the test set. For image preprocessing, the voxel size of the 3D CT images in the NSCLC radiogenomics dataset was changed (1 mm × 1 mm × 1 mm isotropic). The median volume of the lung cancer was 8,219 mm^3^ (interquartile range: 3,461.5–25,357 mm^3^) in the NSCLC radiogenomics dataset.

### Dataset Generation

**FIGURE 2:**
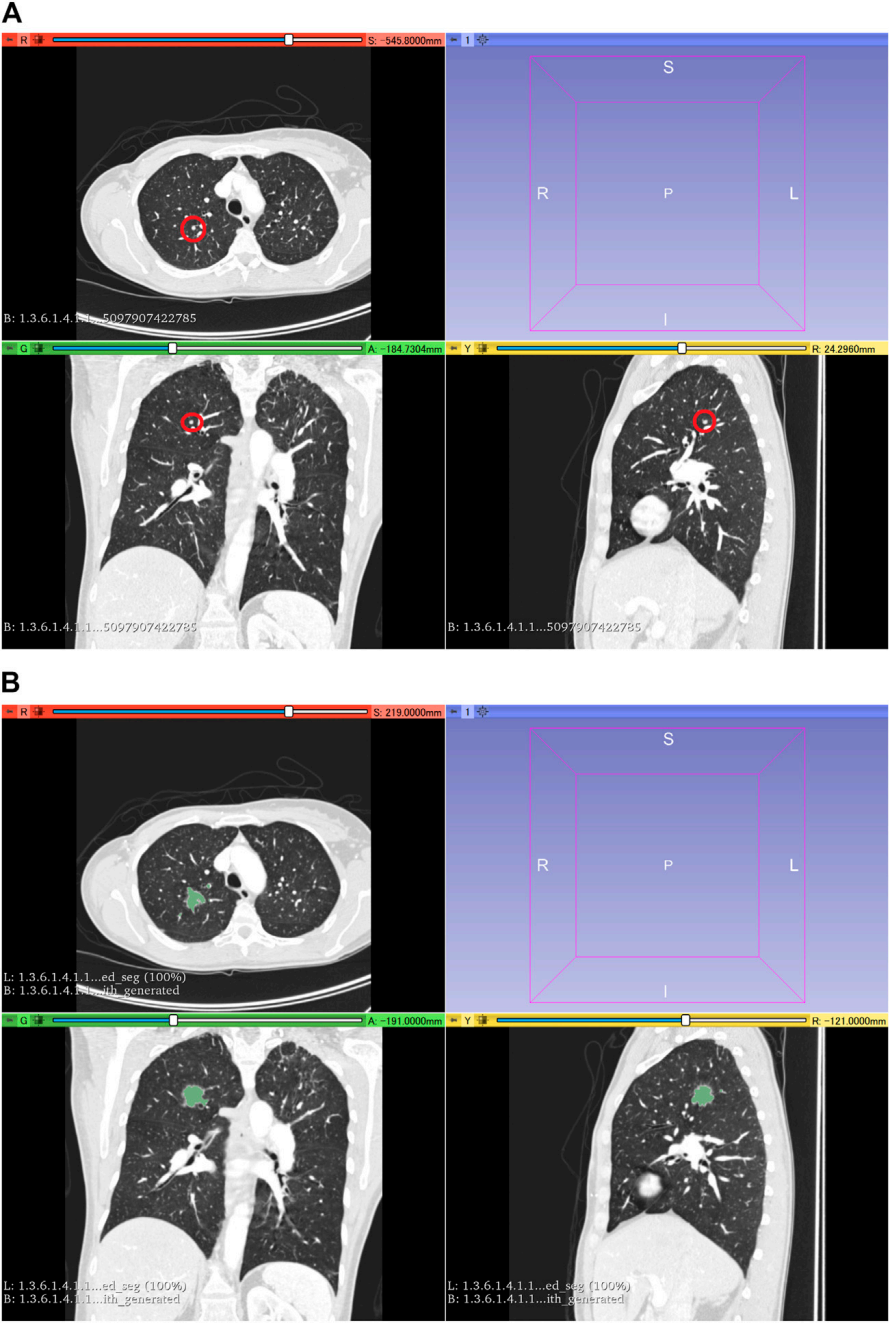
3D CT images of the chest. **(A)** Original CT images in the LUNA16 dataset. The red circle represents the true nodule specified in the LUNA16 dataset. **(B)** Lung nodule is artificially generated at the location of the true nodule. Label obtained with the 3D graph cut is superimposed on the 3D CT images.

The LUNA16 dataset was used to generate an artificial dataset for segmentation. First, lung segmentation was performed for the chest CT images of the LUNA16 dataset, covering the lungs entirely. A pretrained deep learning model (a variant of U-net (Ronneberger et al., 2015)) was used for the lung segmentation (https://github.com/JoHof/lungmask (Hofmanninger et al., 2020)). Subsequently, 3D images of the nodule were generated using the GAN model, which is based on the variant of 3D pix2pix (Nishio et al., 2020a). While the GAN model can generate lung nodules at any location in the lungs, we used locations of true nodules for nodule generation. In addition, we generated only one nodule per CT scan. To determine the location of the generated lung nodule, one location of true nodule was selected from the annotation of the LUNA16 dataset, for each CT scan. Therefore, the locations of generated lung nodules were fixed (no randomness). The true nodule was replaced with the nodule generated using the GAN model. For the nodule generation, 3D CT images were cropped with a volume of interest of 40 × 40 × 40 voxels for the location of the true nodules, and the cropped images were fed to the GAN model. While the size of the generated lung nodules can be adjusted with the GAN model, the GAN model generated the largest nodule as the model (the generation target size was 3 cm or higher). After nodule generation, the segmentation label was automatically generated using the 3D graph cut and Gaussian mixture (https://github.com/mjirik/imcut) (Jirík et al., 2013). Because the intensity of the seed point on the CT images was used to train the Gaussian mixture model, the center area of the generated images (40 × 40×40 voxels) was specified as seed points of the nodule, and the marginal area of the generated images was specified as seed points of the non-nodule (background). The output of the 3D graph cut was used as the segmentation label of the generated nodule. Next, the generated CT images of the nodule were merged with the original CT images. When merging the CT images of the generated nodules, only the areas that were assigned as lung labels in the lung segmentation were targeted for the merging. The areas of the generated CT images that were assigned as non-lung labels were not merged. Figure 2 shows the representative images of the generated nodules and their labels. In total, 165 lung nodules were generated for the 165 sets of 3D CT images in the LUNA16 dataset.

### Segmentation Model

Open-source software (nnUnet) (Isensee et al., 2018) was used for the deep learning model of lung cancer segmentation, which is available at https://github.com/MIC-DKFZ/nnUNet. nnUnet is a variant of U-net (Ronneberger et al., 2015). Originally, nnUnet was used for the Decathlon datasets (Isensee et al., 2018). Because the original nnUnet has no functionality of transfer learning, we modified the source code of nnUnet. With the modification, nnUnet could use a pretrained model and perform transfer learning. In addition, the number of epochs in the training nnUnet could be changed. Except for these two points, no modifications were made to nnUnet. Dataset splitting (training and validation sets) was performed with the default setting of nnUnet.

First, the generated dataset for segmentation obtained from the LUNA16 dataset was used to construct the pretrained model. Two pretrained models were built: one obtained from 300 epochs of training (PM_300_) and the other obtained from 500 epochs of training (PM_500_). Next, transfer learning using the two pretrained models was performed for the three Decathlon lung datasets (Decathlon_full_, Decathlon_mid_, and Decathlon_small_) using the modified nnUnet. At this stage, no new layer was added to the model. Although several studies used layer freezing in transfer learning (Nishio et al., 2020b), no layers of the pretrained model were frozen in this study. To evaluate the effect of transfer learning, models were constructed without transfer learning (original nnUnet). Here, “original nnUnet” means that the source code of nnUnet was not changed, except for changing the number of epochs. The original nnUnet and its default setting were used for the model construction without transfer learning. In the model training, the epochs were set to 100, 300, and 500. The training of each model was started from epoch 1.

### Evaluation of Segmentation Models

As the test dataset, 144 sets of 3D CT images from the NSCLC radiogenomics dataset were used. For each set, the Dice similarity coefficient (DSC) was used to evaluate the segmentation models. In addition, the Jaccard index (JI), sensitivity (SE), and specificity (SP) were calculated as the evaluation metrics, which is expressed as follows:

$$\begin{array}{c} DSC=\;\frac{2\left|P\cap L\right|}{\left|P\right|+\left|L\right|}, \end{array}$$ (1)

$$\begin{array}{c} JI=\;\frac{\left|P\cap L\right|}{\left|P\right|+\left|L\right|-\left|P\cap L\right|}, \end{array}$$ (2)

$$\begin{array}{c} SE=\;\frac{\left|P\cap L\right|}{\left|L\right|}, \end{array}$$ (3)

$$\begin{array}{c} SP=\;\frac{\left|I\right|-\left(\left|L\right|+\left|P\right|-\left|P\cap L\right|\right)}{\left|I\right|-\left|L\right|}, \end{array}$$ (4)

where |*P*|, |*L*|, and |*I*| denote the number of voxels for the segmentation results, label of the lung cancer segmentation, and 3D CT images, respectively. |*P* ∩ *L*| represents the number of voxels where nnUnet can accurately segment the lung cancer (true positive). Before calculating the four metrics, a threshold of 0.5 was used to obtain the segmentation mask from the output of nnUnet.

Differences of DSC were statistically tested with the Wilcoxon signed rank test. To control the family-wise error rate, the Bonferroni correction was used; *p*-values less than 0.01666 were considered statistically significant. Statistical analyses were performed using R (version 4.0.4, https://www.r-project.org/).

## Results

**FIGURE 3:**
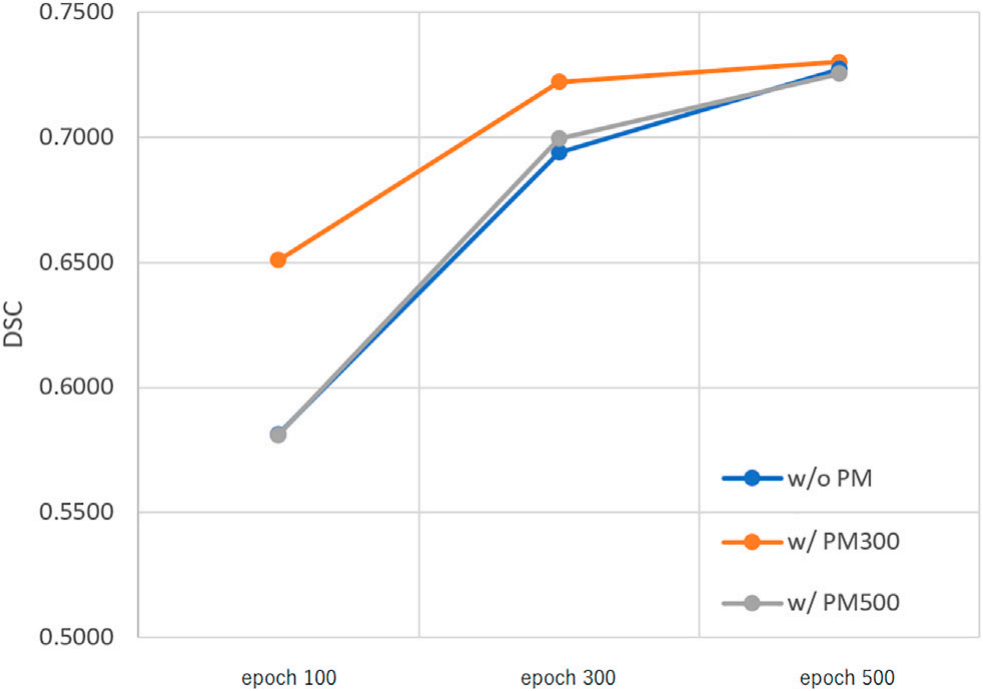
Mean DSC of the test set when using Decathlon_full_. Abbreviation: PM, pretrained model; PM_300_, pretrained model obtained from 300 epochs of training; PM_500_, pretrained model obtained from 500 epochs of training.

**FIGURE 4:**
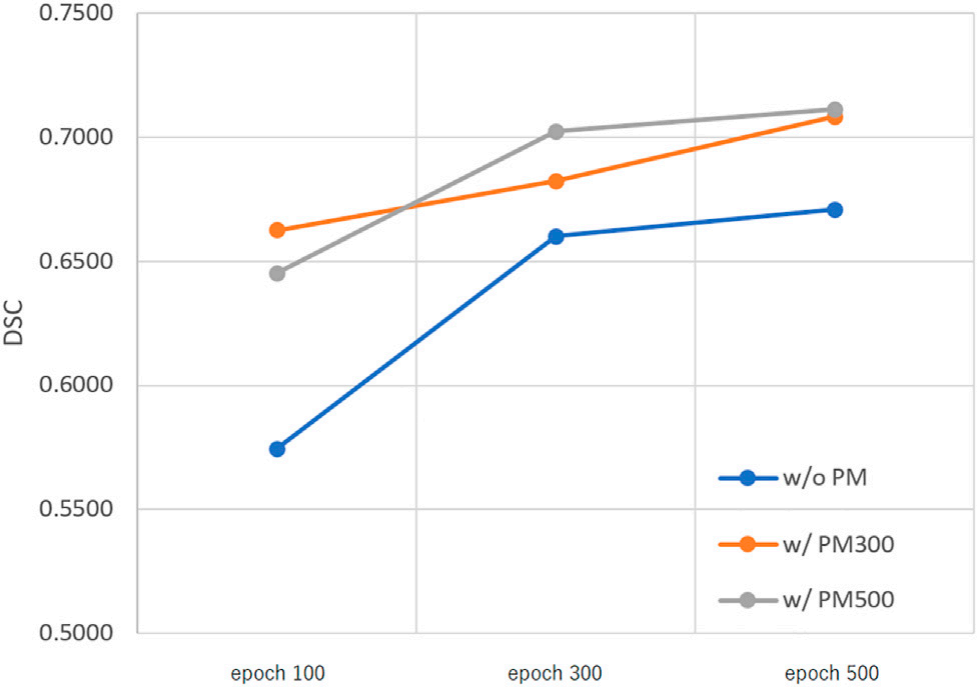
Mean DSC of the test set when using Decathlon_mid_. Abbreviation: PM, pretrained model; PM_300_, pretrained model obtained from 300 epochs of training; PM_500_, pretrained model obtained from 500 epochs of training.

**FIGURE 5:**
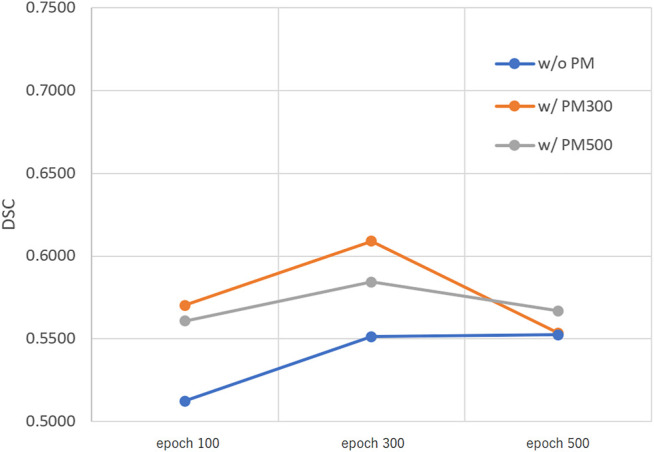
Mean DSC of the test set when using Decathlon_small_. Abbreviation: PM, pretrained model; PM_300_, pretrained model obtained from 300 epochs of training; PM_500_, pretrained model obtained from 500 epochs of training.

Figures 3–5 show the mean DSC of the test set with and without PM_300_ and PM_500_ when Decathlon_full_, Decathlon_mid_, and Decathlon_small_ are used as the training sets, respectively. In these figures, the results without PM correspond to those of original nnUnet. Generally, PM_300_ and PM_500_ improved the mean DSC of nnUnet, compared with the original nnUnet (without the pretrained model). In particular, the effectiveness of the pretrained model was high when using Decathlon_mid_ as the training set. Neither PM_300_ nor PM_500_ was useful for DSC improvement when Decathlon_full_ and Decathlon_small_ were used in the 500-epoch training. The DSC improvement was greater in the 100 and 300 epochs than that in the 500 epochs.

Tables 2–4 list the mean and standard deviation of the four metrics of the test set with and without PM_300_ and PM_500_ when Decathlon_full_, Decathlon_mid_, and Decathlon_small_ are used as the training sets, respectively. Because the volume ratio between cancer and noncancerous regions is extremely low, SP was extremely high in the current study. Regarding DSC, JI, and SE, the same trend can be observed. PM_300_ and PM_500_ improved the mean values of the three metrics; improvement in JI and SE was greater in the 100 and 300 epochs than that in the 500 epochs. Table 5 shows *p*-values for differences of DSC in Decathlon_full_, Decathlon_mid_, and Decathlon_small_.

**TABLE 2:** Segmentation metrics of test set using Decathlon_full_.

Model	DSC mean	SD	JI mean	SD	SE mean	SD	SP mean	SD
W/o PM, epoch 100	0.5813	0.2495	0.4505	0.2352	0.7049	0.2514	0.99984	0.00027
W/PM_300,_ epoch 100	0.6510	0.2104	0.5150	0.2110	0.7672	0.1947	0.99987	0.00024
W/PM_500,_ epoch 100	0.5810	0.2474	0.4510	0.2411	0.7511	0.2133	0.99982	0.00020
W/o PM, epoch 300	0.6939	0.2409	0.5741	0.2360	0.7371	0.2542	0.99992	0.00011
W/PM_300,_ epoch 300	0.7221	0.2055	0.5995	0.2172	0.7580	0.2102	0.99991	0.00023
W/PM_500,_ epoch 300	0.6995	0.2302	0.5789	0.2347	0.7514	0.2119	0.99992	0.00012
W/o PM, epoch 500	0.7273	0.2266	0.6120	0.2315	0.7334	0.2464	0.99995	0.00009
W/PM_300,_ epoch 500	0.7301	0.2145	0.6122	0.2239	0.7662	0.2061	0.99990	0.00025
W/PM_500,_ epoch 500	0.7255	0.2424	0.6139	0.2386	0.7428	0.2484	0.99993	0.00012

Abbreviations: DSC, Dice similarity coefficients; JI, Jaccard index; SE, sensitivity; SP, specificity; PM, pretrained model; PM300, pretrained model obtained from 300 epochs of training; PM_500_, pretrained model obtained from 500 epochs of training.

**TABLE 3:** Segmentation metrics of test set using Decathlon_mid_.

Model	DSC mean	SD	JI mean	SD	SE mean	SD	SP mean	SD
W/o PM, epoch 100	0.5744	0.2704	0.4493	0.2486	0.6122	0.2830	0.99993	0.00008
W/PM_300,_ epoch 100	0.6624	0.2305	0.5342	0.2303	0.6999	0.2221	0.99992	0.00012
W/PM_500,_ epoch 100	0.6452	0.2351	0.5155	0.2302	0.7144	0.2302	0.99990	0.00015
W/o PM, epoch 300	0.6600	0.2572	0.5398	0.2509	0.6750	0.2579	0.99993	0.00013
W/PM_300,_ epoch 300	0.6822	0.2470	0.5631	0.2463	0.7234	0.2294	0.99991	0.00015
W/PM_500,_ epoch 300	0.7024	0.2308	0.5823	0.2344	0.7292	0.2201	0.99990	0.00022
W/o PM, epoch 500	0.6708	0.2689	0.5570	0.2629	0.6892	0.2608	0.99991	0.00019
W/PM_300,_ epoch 500	0.7083	0.2039	0.5819	0.2160	0.7479	0.1936	0.99992	0.00012
W/PM_500,_ epoch 500	0.7112	0.2228	0.5909	0.2293	0.7266	0.2215	0.99993	0.00013

Abbreviations: DSC, Dice similarity coefficients; JI, Jaccard index; SE, sensitivity; SP, specificity; PM, pretrained model; PM_300_, pretrained model obtained from 300 epochs of training; PM_500_, pretrained model obtained from 500 epochs of training.

**TABLE 4:** egmentation metrics of test set using Decathlon_small_.

Model	DSC mean	SD	JI mean	SD	SE mean	SD	SP mean	SD
W/o PM, epoch 100	0.5124	0.2857	0.3928	0.2555	0.4671	0.2838	0.99997	0.00007
W/PM_300,_ epoch 100	0.5702	0.2513	0.4400	0.2388	0.5183	0.2524	0.99996	0.00007
W/PM_500,_ epoch 100	0.5608	0.2681	0.4352	0.2491	0.5190	0.2761	0.99997	0.00006
W/o PM, epoch 300	0.5515	0.2742	0.4274	0.2510	0.4950	0.2811	0.99997	0.00007
W/PM_300,_ epoch 300	0.6090	0.2414	0.4781	0.2361	0.5511	0.2510	0.99997	0.00007
W/PM_500,_ epoch 300	0.5844	0.2607	0.4574	0.2466	0.5215	0.2719	0.99997	0.00006
W/o PM, epoch 500	0.5525	0.2855	0.4316	0.2573	0.5109	0.2851	0.99997	0.00006
W/PM_300,_ epoch 500	0.5536	0.2856	0.4338	0.2629	0.4934	0.2878	0.99998	0.00005
W/PM_500,_ epoch 500	0.5670	0.2731	0.4435	0.2557	0.5089	0.2786	0.99997	0.00006

Abbreviations: DSC, Dice similarity coefficients; JI, Jaccard index; SE, sensitivity; SP, specificity; PM, pretrained model; PM_300_, pretrained model obtained from 300 epochs of training; PM_500_, pretrained model obtained from 500 epochs of training.

**TABLE 5:** *p*-values for differences of DSC in Decathlon_full_, Decathlon_mid_, and Decathlon_small_.

Pair	Decathlon_full_	Decathlon_mid_	Decathlon_small_
Epoch 100	—	—	—
w/o PM vs. w/PM_300_	7.857 × 10^−5^	1.877 × 10^−7^	0.0006931
w/o PM vs. w/PM_500_	0.9682	0.0001075	0.0009121
w/o PM_300_ vs. w/PM_500_	1.606 × 10^−5^	0.4642	0.1499
Epoch 300	—	—	—
w/o PM vs. w/PM_300_	0.2346	0.04381	6.662 × 10^−5^
w/o PM vs. w/PM_500_	0.7664	0.000649	0.001194
w/o PM_300_ vs. w/PM_500_	0.1493	0.01325	0.3050
Epoch 500	—	—	—
w/o PM vs. w/PM_300_	0.9743	0.2322	0.5990
w/o PM vs. w/PM_500_	0.7243	0.005435	0.8746
w/o PM_300_ vs. w/PM_500_	0.9534	0.06595	0.9229

Abbreviations: DSC, Dice similarity coefficients; PM, pretrained model; PM_300_, pretrained model obtained from 300 epochs of training; PM_500_, pretrained model obtained from 500 epochs of training.

**FIGURE 6:**
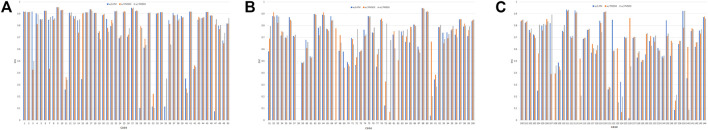
DSC values of the test set when using Decathlon_mid_ with and without the pretrained model. **(A)** Cases 1–50, **(B)** cases 51–100, and **(C)** cases 101–144. Note: DSC values are obtained with models obtained from 500 epochs of training. Abbreviation: PM, pretrained model; PM_300_, pretrained model obtained from 300 epochs of training; PM_500_, pretrained model obtained from 500 epochs of training.

**FIGURE 7:**
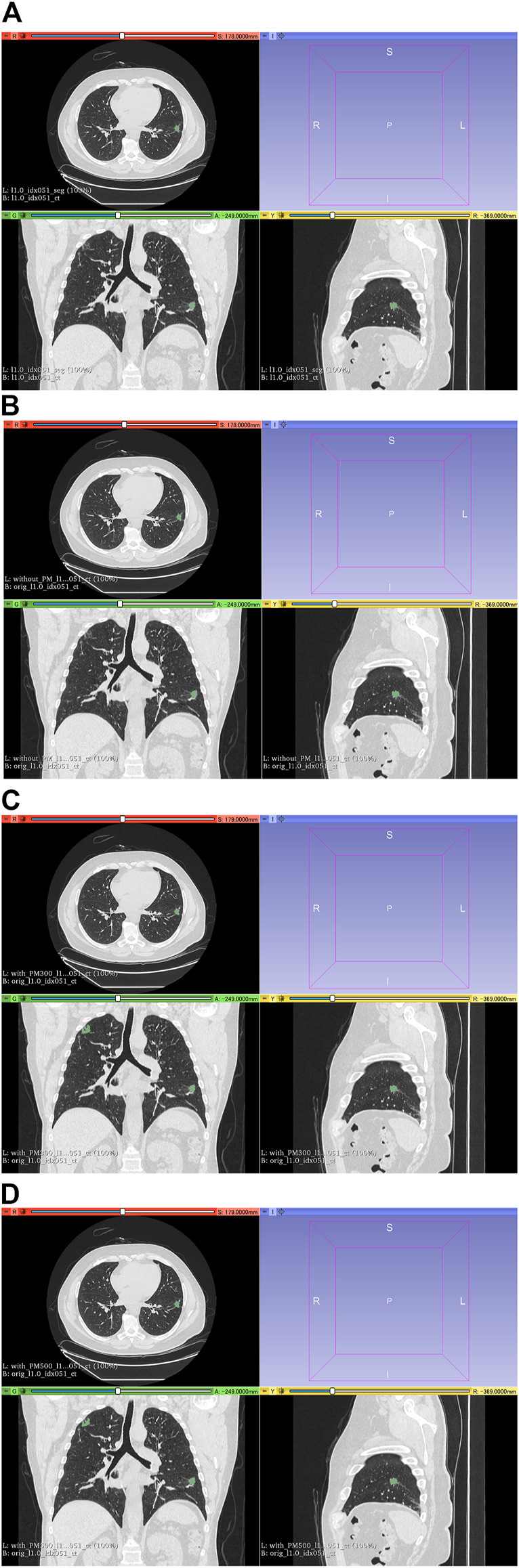
Results of segmentation in case 3 of test set. **(A)** CT images and ground-truth labels. **(B)** CT images and segmentation results without PM. **(C)** CT images and segmentation results with PM_300_. **(D)** CT images and segmentation results with PM_500_. Note: Because of PM, a part of the right upper field is incorrectly segmented as lung cancer in **(C)** and **(D)**. Abbreviation: PM, pretrained model; PM_300_, pretrained model obtained from 300 epochs of training; PM_500_, pretrained model obtained from 500 epochs of training.

**FIGURE 8:**
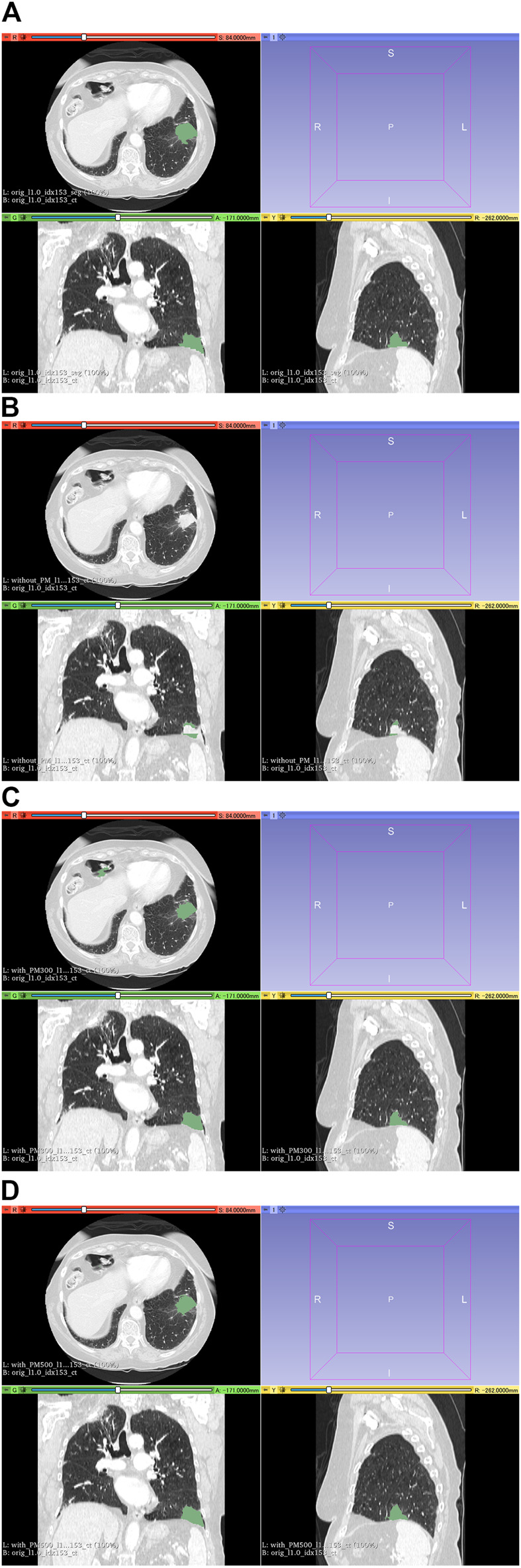
Results of segmentation in case 104 of the test set. **(A)** CT images and ground-truth labels. **(B)** CT images and segmentation results without PM. **(C)** CT images and segmentation results with PM_300_. **(D)** CT images and segmentation results with PM_500_. Note: With the aid of PM, lung cancer is correctly segmented in **(C)** and **(D)**. Abbreviation: PM, pretrained model; PM_300_, pretrained model obtained from 300 epochs of training; PM_500_, pretrained model obtained from 500 epochs of training.

Figure 6 shows all the DSC values of the test set when using Decathlon_mid_ with and without the pretrained model. Figures 7, 8 show the representative segmentation results. Figures 7, 8 show the CT images in which PM is ineffective and effective, respectively. Supplementary Table 1 includes the segmentation results when the generated dataset consisted of variable-size–generated nodules. In addition, the Supplementary Table 2 includes visual evaluation results of cases with low DSC values.

## Discussion

In this study, we proposed a pretrained model for segmentation constructed from an artificial dataset of lung nodules generated using the GAN and 3D graph cut. Our results show that the accuracy of lung cancer segmentation could be improved when this pretrained model was used for transfer learning in the segmentation process. The effectiveness of the pretrained model was higher on the Decathlon_mid_ and Decathlon_small_ datasets than that of the pretrained model on the Decathlon_full_ dataset, suggesting that our proposed method may be effective on small datasets.

The pretrained model was more effective when the number of training epochs was low. In other words, the number of epochs required to achieve a sufficient segmentation performance was lower with the pretrained model than without it. This may be attributed to the fact that the pretrained model provides good initial values for the trainable parameters of nnUnet.

Previously, a study used U-net and GAN combinedly for multi-organ segmentation on 3D CT images (Dong et al., 2019). However, the study did not use a pretrained model. Another study was conducted on a classification model using a dataset generated with GANs and a pretrained model (Onishi et al., 2020). To the best of our knowledge, no studies have been reported on segmentation models with GANs and a pretrained model. Our results and those of Onishi et al. (2020) indicate that the GAN generated dataset, and its pretrained models may be useful for various tasks.

Several studies have reported the use of artificially generated datasets using the GAN for data augmentation (Jin et al., 2018; Onishi et al., 2019; Yang et al., 2019; Muramatsu et al., 2020). Similarly, in this study, we tried to use a dataset generated using the GAN for data augmentation. However, we could not obtain effective results for lung cancer segmentation when the artificial dataset was used as data augmentation (data not shown in this article). Instead, we constructed a pretrained model for the segmentation using the generated lung nodules and performed transfer learning based on the pretrained model, yielding higher lung cancer segmentation accuracy. Although it was difficult to perform accurate classification between the generated lung nodules and the true lung nodules (Nishio et al., 2020a), the generated lung nodules had little variation as lung cancer. It is speculated that mixing the generated lung nodules with the true lung nodules could distort the distribution as the dataset of lung cancer segmentation and adversely affect the model training of nnUnet.

Generally, supervised learning (e.g., nnUnet) requires annotation data as the dataset. On the datasets of lung cancer segmentation, clinicians frequently annotate lung cancer on CT images to build lung cancer datasets, which is time consuming and labor intensive. Although it is possible to manually annotate the generated data of our dataset, we decided to use the 3D graph cut to obtain annotation data of the generated lung nodules. This made it possible to build an artificial dataset for the segmentation without requiring any manual task.

Although the generated lung nodules and the pretrained model based on them could effectively improve the accuracy of lung cancer segmentation, this pretrained model is not always effective. For example, the effectiveness of the pretrained model was not observed in the 500-epoch training of Decathlon_full_ and Decathlon_small_. For the former case, this was attributed to the fact that Decathlon_full_ had sufficient amount of data and the number of training epochs was high. In the latter, the number of datasets was very small (10 cases). Therefore, even when the pretrained model was used, the training segmentation model was unstable, and the effectiveness of the pretrained model was limited.

Our study has some limitations. First, we used three public datasets containing images of lung nodules and/or lung cancer. However, we did not verify whether the generalizability of our segmentation model can be improved under external variation. Second, we focused on lung nodules and/or lung cancer in the current study. Therefore, the effectiveness of our method for other diseases or other organs has not been validated. In particular, it is necessary to confirm whether the automatic generation of annotation data using the 3D graph cut can be applied to other diseases and other organs. Third, because of the GAN model’s limitation (Nishio et al., 2020a), it was impossible to generate lung nodules larger than 40 mm. Therefore, the effect of large generated nodules is not investigated in the current study.

In conclusion, the proposed method comprising an artificial dataset and a pretrained model can improve the accuracy of lung cancer segmentation; however, it should be further investigated for other diseases and other organs.

## Data Availability

Publicly available datasets were analyzed in this study. These data can be found here: 1. https://luna16.grand-challenge.org/ 2. http://medicaldecathlon.com/ 3. https://wiki.cancerimagingarchive.net/display/Public/NSCLC-Radiomics.
