# Supplementary material for: Lung Cancer Segmentation With Transfer Learning: Usefulness of a Pretrained Model Constructed From an Artificial Dataset Generated Using a Generative Adversarial Network
Source: Front Artif Intell. 2021 Jul 16;4:694815. doi: 10.3389/frai.2021.694815 (PMC8322116; doi:10.3389/frai.2021.694815)
Supplement: Supplementary file 3 [file DataSheet1.docx]

Supplementary Material

# Additional experiment for investigating effect of nodule size variability

## Method

To evaluate effect of nodule size variability, an artificial dataset was generated using LUNA16 and 3D GAN. For the 165 sets of CT images of LUNA16 dataset used in the main experiments, lung nodules were generated to prepare the artificial dataset for the additional experiment. Because of the implementation of 3D GAN, the following sizes were used as generation target size: (i) 10–20 mm, (ii) 20–30 mm, and (iii) more than 30 mm. Except generation target size, the same processing was used. In this additional experiment, generation target size was randomly selected from (i)–(iii). After the nodule generation, model training and segmentation performance evaluation were performed as described in the main text.

## Results (Supplementary Table 1)

Supplementary Table 1 is an MS Excel file. Supplementary Table 1 includes results of effect of nodule size variability. In addition, values of Tables 2–4 of main manuscript are included in this Table. The results of model pretrained with variable-size nodules are indicated with “variable”.

DSC, JI, SE, SP are used as segmentation metric in Supplementary Table 1.

Mean, SD, skewness, kurtosis, mode, 1^st^ quartile, and 3^rd^ quartile are used as descriptive statistic in Supplementary Table 1.

## Discussion

According to Supplementary Table 1, nodule size variability is effective in some models. For example, model “w/ PM300_variable, epoch 100” achieved the high value of DSC in epoch = 100 using Decathlon_full_. However, differences of DSC with and without nodule size variability are small in some models, especially using Decathlon_small_. This may be caused by the fact that relatively small nodules were generated in the additional experiment. Because the median volume of the lung cancer was 8219 mm^3^ in the NSCLC Radiogenomics dataset, majority of lung cancer is larger than 20 mm (if lung cancer is sphere and size is 20 mm, its volume = pi x 4 / 3 x 10 x 10 x 10 = 4187 mm^3^). Since small lung cancer is minor in the NSCLC Radiogenomics dataset, effectiveness of small generated nodule was limited in DSC of the test set.

# Visual evaluation for cases with low DSC values (Supplementary Table 2)

Based on Figure 6 of the main manuscript, 25 cases had low DSC values among the 144 cases of test set. These cases were visually evaluated by the board-certified radiologist with 15-year experience.

Based on the visual evaluation, the reason for the low values of DSC is described in Supplementary Table 2. Supplementary Table 2 is an MS Excel file.
